# A single-center, open-labeled, randomized, 6-month, parallel-group study to assess the safety and efficacy of allogeneic cultured keratinocyte sheet transplantation for deep second-degree burn wounds: rationale and design of phase I/II clinical trial

**DOI:** 10.1186/s13063-024-08070-4

**Published:** 2024-04-01

**Authors:** Shayan Farzanbakhsh, Mohammad Amin Shahrbaf, Hoda Madani, Mostafa Dahmardei, Bahareh Sadri, Massoud Vosough

**Affiliations:** 1 Department of Regenerative Medicine, Cell Science Research Center, Royan Institute for Stem Cell Biology and Technology, ACECR, Tehran, Iran; 2 Department of Plastic & Reconstructive Surgery, School of Medicine, Stem Cell and Regenerative Medicine Research Center, Shahid Motahari Burns Hospital, Iran University of Medical Sciences, Tehran, Iran

**Keywords:** Skin substitute, Burns, Wound closure, Allograft, Keratinocyte

## Abstract

**Background:**

Burn-related injuries are a major global health issue, causing 180,000 deaths per year. Early debridement of necrotic tissue in association with a split-thickness skin graft is usually administered for some of the 2nd- and 3rd-degree injuries. However, this approach can be complicated by factors such as a lack of proper donor sites. Artificial skin substitutes have attracted much attention for burn-related injuries. Keratinocyte sheets are one of the skin substitutes that their safety and efficacy have been reported by previous studies.

**Methods:**

Two consecutive clinical trials were designed, one of them is phase I, a non-randomized, open-label trial with 5 patients, and phase II is a randomized and open-label trial with 35 patients. A total number of 40 patients diagnosed with 2nd-degree burn injury will receive allogenic keratinocyte sheet transplantation. The safety and efficacy of allogeneic skin graft with autograft skin transplantation and conventional treatments, including Vaseline dressing and topical antibiotic, will be compared in different wounds of a single patient in phase II. After the transplantation, patients will be followed up on days 3, 7, 10, 14, 21, and 28. In the 3rd and 6th months after the transplantation scar, a wound closure assessment will be conducted based on the Vancouver Scar Scale and the Patient and Observer Scar Assessment Scale.

**Discussion:**

This study will explain the design and rationale of a cellular-based skin substitute for the first time in Iran. In addition, this work proposes this product being registered as an off-the-shelf product for burn wound management in the country.

**Trial registration:**

Iranian Registry of Clinical Trials (IRCT) IRCT20080728001031N31, 2022-04-23 for phase I and IRCT20080728001031N36, 2024-03-15 for phase II.

## Administrative information

Note: The numbers in curly brackets in this protocol refer to the SPIRIT checklist item numbers. The order of the items has been modified to group similar items (see http://www.equator-network.org/reporting-guidelines/spirit-2013-statement-defining-standard-protocol-items-for-clinical-trials/).

**Table:** 

Title {1}	A single-center, open-labeled, randomized, 6-month, parallel-group study to assess the safety and efficacy of allogeneic cultured keratinocyte sheet transplantation for deep second-degree burn wounds: rationale and design of phase I/II clinical trial
Trial registration {2a and 2b}	IRCT: IRCT20080728001031N31, IRCT20080728001031N36
Protocol version {3}	This is protocol version 1 designed on 2022–04-25.
Funding {4}	The keratinocyte sheet was made in the Advanced Therapy Medicinal Product Technology (ATMP) center at Royan Institute. This work has received grants from the Royan Institute for Stem Cell Biology and Technology (ID: RI.400000193). The funding sources had no responsibilities in the study design; data collection, analysis, and interpretation; manuscript writing; and decision to submit this paper for publication.
Author details {5a}	S.F [Department of Regenerative Medicine, Cell Science Research Center, Royan Institute for stem cell biology and technology, Tehran, Iran.], M.A.S [Department of Regenerative Medicine, Cell Science Research Center, Royan Institute for stem cell biology and technology, Tehran, Iran.], H.M [Department of Regenerative Medicine, Cell Science Research Center, Royan Institute for stem cell biology and technology, Tehran, Iran.], M.D [Department of Plastic & Reconstructive Surgery, School of Medicine, Stem Cell and Regenerative Medicine Research Center, Shahid Motahari Burns Hospital, Iran University of Medical Sciences, Tehran, Iran.], B.S [Department of Regenerative Medicine, Cell Science Research Center, Royan Institute for stem cell biology and technology, Tehran, Iran.], M.V [Department of Regenerative Medicine, Cell Science Research Center, Royan institute for stem cell biology and technology, Tehran, Iran.] S.F. and M.A.S. reviewed the literature and drafted the manuscript. S.F., M.A.S., H.M., M.D., and B.S. participated in the study design and setting of the protocol. H.M. and B.S. reviewed the manuscript. M.V. conceived the design of the study and reviewed and confirmed the final manuscript. All authors contributed to the refinement of the study protocol and approved the final manuscript.
Name and contact information for the trial sponsor {5b}	Trial sponsor: Royan Institute Sponsor’s reference: RI400000193 Contact name: Dr. Massoud Vosough Address: Department of Regenerative Medicine, Cell Science Research Center, Royan Institute for Stem Cell Biology and Technology, ACECR, Tehran, Iran. Telephone: + 989,121,196,454 Email: masvos@royaninstitute.org
Role of sponsor {5c}	The sponsor had no responsibilities in the study design; data collection, analysis, and interpretation; manuscript writing; and decision to submit this paper for publication.

## Introduction

### Background and rationale {6a}

Burn-related injuries are a major global health issue, causing 180,000 deaths per year, and are most commonly observed in low- and middle-income countries [1]. These injuries are not always associated with mortality but also can cause complications including infection, multiorgan failure, and aesthetic outcomes like hypertrophic scars or keloid growth in some non-fatal cases, usually resulting in long-term hospitalization and disability [2]. Burn injuries can cause physical and mental complications for the patients and lead to poor quality of life [3]. In addition, the cost of treating burn injuries is high and increases in association with the percentage of the total injured area [4].

Several etiologies can cause burn-related injuries, such as fire, explosives, electricity, and hazardous chemical substances [5]. Certain occupations dealing with flammable materials, being a smoker, alcohol abusers, and lack of safety in household places are some risk factors that increase the likelihood of burn injuries [6]. According to the global assessment of burn injuries from 1990 to 2019, the age-standardized rates of burn incidence and the mortality rate were decreased, whereas the number of new burn cases had an increasing trend during the last three decades [7].

### Burn injury classification

Classification of burn injuries is crucial for optimal assessment and effective management [8]. Factors related to the severity of a burn injury include the anatomical location of the affected site, the temperature degree, the duration of exposure, and the cause of the injury [9]. Burn injuries can be classified into four degrees based on the progression in different layers of the skin, size, and the depth of the injury [10]. First-degree burns are specified by the involvement of the epidermis layer, which are not emergencies and can be self-limited with proper care within less than a week. In the context of superficial or deep involvement of the dermis, the burn injury is classified as a second-degree injury, associated with scar development in severe cases, and surgical intervention may be needed in some severe cases [11]. Third-degree burn injuries are usually painless, with involvement of the entire skin, and require surgery [12]. In the case of deep tissue damage including muscles and bones, the burn injury is fourth-degree, which usually leads to the loss of the burned limb [13].

### Treatment challenges

Although the skin has the ability to heal itself, severe burns require a range of interventions, such as healing medications, debridement, and skin grafts [14]. Early debridement of necrotic tissue in association with a split-thickness skin graft (STSG), consisting of the epidermis and a small part of the underlying dermis, is usually administered for some 2nd- and 3rd-degree injuries as a standard therapeutic method [15]. However, this approach can be complicated by factors such as lack of proper donor sites, low-quality skin graft, low vascularity, and hematoma formation [16]. In addition, the development of scar tissue and focal contracture can lead to a substantial reduction in joint activity and even loss of function [17].

### Novel therapies

Nowadays, thanks to the advancements in molecular and personalized medicine, the management of burn injuries entered a new era [18]. To date, many progressions have been conducted to ameliorate burn wounds, which facilitates different stages of wound healing including ischemic phase, inflammatory phase, or wound rehydration [19]. Regenerative medicine and stem cell-based treatments have had promising advancements in the management of different types of wounds through accelerating the healing process [20]. The administration of regenerative medicine for burn injuries offered innovative and effective therapies for affected patients [21]. Artificial skin substitutes are developed as advanced medicinal products and have attracted much attention for burn-related injuries [22]. Skin substitutes prevent electrolyte loss, which is one of the major causes of morbidity and mortality in burn injuries. In addition, these skin substitutes have some advantages in comparison with the surgical skin grafts, such as lower infection rate, faster healing process, and less scar tissue [23, 24]. Keratinocyte sheets are one of the skin substitutes that their safety and efficacy have been reported by previous studies [25, 26]. For instance, Kim et al. investigated the effectiveness of the keratinocyte sheet (Kaloderm®) in deep second-degree burn wounds and compared outcomes with standard treatments (chlorhexidine gauze and antibiotic ointment) as control [26].

### Objectives {7}

In phase I of this clinical trial, we are going to design an open-label study to assess the safety and feasibility of the keratinocyte sheet, made by Royan Institute. In phase II of this study, we will assess the safety and efficacy of this product by evaluation of time to wound epithelialization, skin graft take rate, scar assessment, wound closure, and quality of skin structure.

### Trial design {8}

Two consecutive clinical trials were designed, one of them is a phase I, non-randomized, open-label trial, and phase II is a randomized and open-label trial. The second phase will be started after the last follow-up of phase I study, if no SAEs related to the transplantation of keratinocyte sheet is reported. In these studies, we will evaluate the safety and efficacy of allogeneic cultured keratinocyte sheet transplantation for healing deep second-degree burn wounds as well as the superiority of this product to conventional treatments.

## Methods: participants, interventions, and outcomes

### Study setting {9}

This study will be conducted at Shahid Motahari Hospital, Iran University of Medical Sciences, Tehran, Iran. Shahid Motahari Hospital is one of the burn referral hospitals in Iran. Therefore, the composition of the population referring to this hospital shows the composition of burn patients in the whole country.

### Eligibility criteria {10}

Prior to the commencement of any study procedures, patients are required to sign the written informed consent.

The following are the inclusion criteria:

1. Age between 18 and 70 years
2. Both genders
3. Acute second-degree burn injuries involving 20–50% of the total body surface area (TBSA)
4. At least one burn injury for phase 1 and three burn injuries for phase 2 with a maximum area of 60 cm^2^ in the trunk or limbs, with intact musculoskeletal tissue

The following are the exclusion criteria:

1. Having underlying diseases which affect wound healing such as cardiovascular disease, diabetes, malnutrition, and hypertension
2. Pregnancy or breastfeeding
3. Active infection, malignancy, or immunosuppression
4. Respiratory tract injury
5. Having additional trauma injury such as fracture or CNS trauma
6. Need for intensive care
7. Unwillingness to cooperate in the intervention and follow-ups

### Who will take informed consent? {26a}

All participants will sign informed consents after an accurate explanation of the process by the physician (S.F.)

### Additional consent provisions for collection and use of participant data and biological specimens {26b}

N/a. All necessary items have been mentioned in the main informed consent, and there is not any ancillary study.

### Interventions

#### Explanation for the choice of comparators {6b}

Autologous skin transplantation or conventional treatment with Vaseline gauze and antibiotics have been considered as comparators due to the fact that these therapeutic methods are the most common standard treatments.

#### Intervention description {11a}

**Fig. 1:**
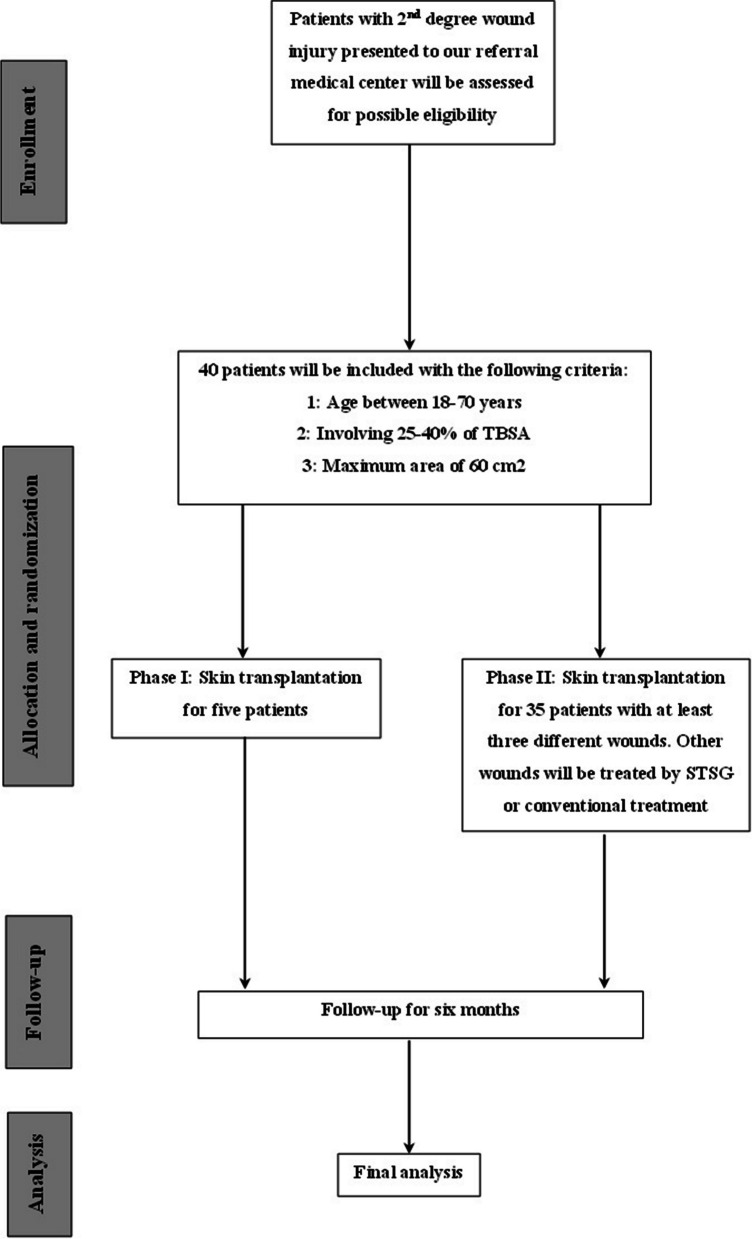
Patients flow diagram phase I and II

**Fig. 2:**
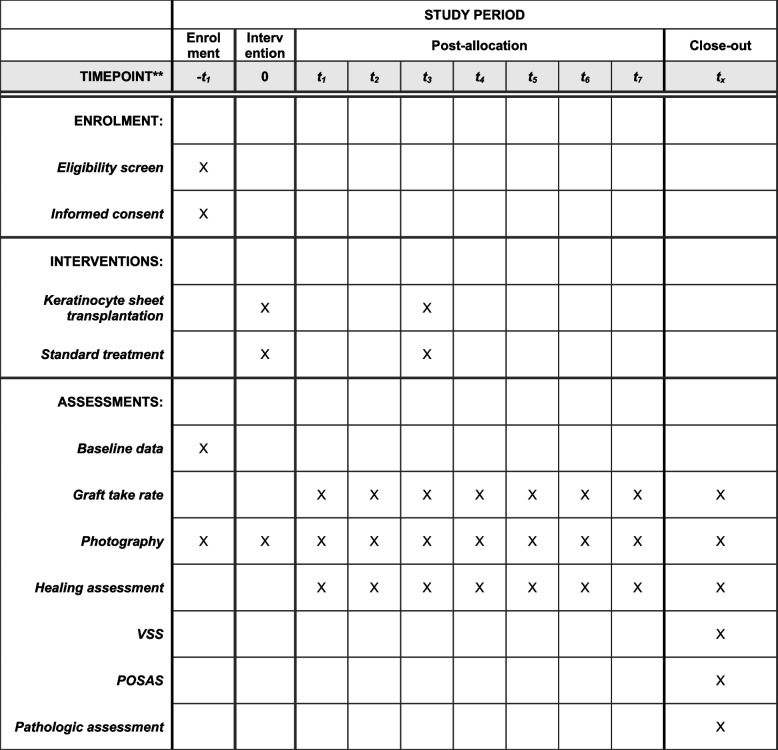
SPIRIT flowchart of phase I

**Fig. 3:**
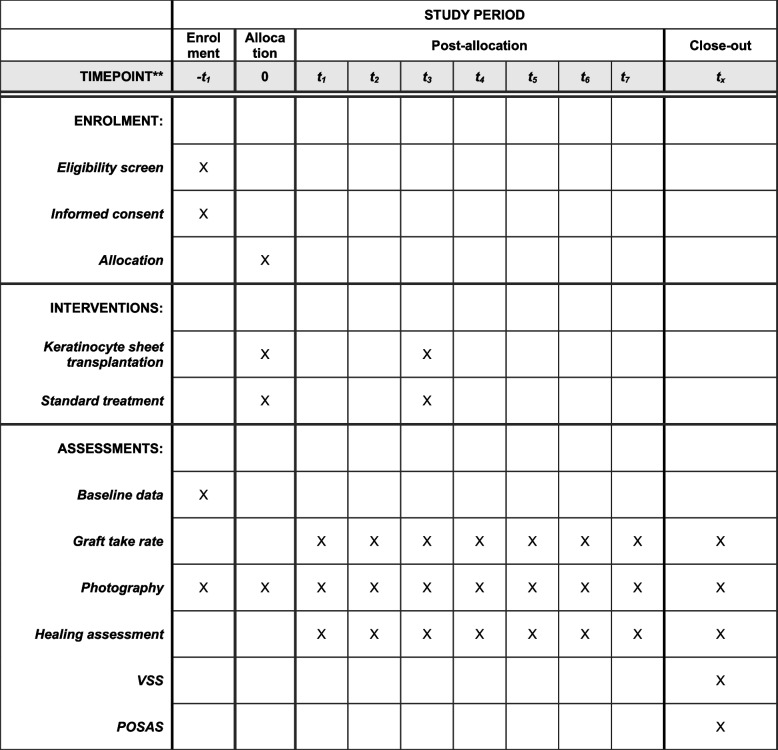
SPIRIT flowchart of phase II

In the first phase of the study, following initial resuscitation, patients will receive allogenic transplantation within the first 5–10 days after the injury. After preparing the patient for the surgery and through general anesthesia in the operating room of Shahid Motahari Hospital, Tehran, Iran, debridement of the necrotic tissues will be conducted by the tangential method. After that, an adrenaline-soaked gauze will be placed on the wound site for 3–5 min to control the bleeding. After preparing the underlying condition, a 2nd-degree burn wound with a maximum area of 60 cm^2^ will be selected and will be covered by an allogeneic keratinocyte sheet. In the second phase of this project, the same procedure will be conducted; however, based on the randomization list, three separate wounds of a single patient will be selected, and allogeneic keratinocyte sheet transplantation, autologous skin transplantation, or conventional treatment will be selected for each wound. Encountering any serious adverse events (SAEs), the second transplantation of the keratinocyte sheet will not be administrated and the patient who experienced SAEs will receive conventional treatment. Figure 1 provides a flow diagram for phases I and II of this protocol. In addition, the SPIRIT flowcharts of the study are presented in Figs. 2 and 3.

#### Criteria for discontinuing or modifying allocated interventions {11b}

Once any reports of SAEs related to the product transplantation are documented, this trial will be terminated.

#### Strategies to improve adherence to interventions {11c}

The patients will be monitored by regular follow-ups on days 3, 7, 10, 14, 21, and 28, after the transplantation. In addition, in the 3rd and 6th month after the transplantation, we will also have additional follow-up sessions which could provide an excellent adherence to the intervention.

#### Relevant concomitant care permitted or prohibited during the trial {11d}

During the study, the patients are allowed to use medications such as non-steroidal anti-inflammatory drugs (NSAIDs) for symptom alleviation.

#### Provisions for post-trial care {30}

N/a. There is no ancillary study and post-trial care. The patients in phases 1 and 2 will be followed up for 6 months after the first transplantation.

### Outcomes {12}

The primary endpoint for this study is safety, which will be evaluated in all follow-ups by Common Terminology Criteria for Adverse Event (CTCAE), version 5.0 [27]. Furthermore, secondary endpoints of this project are time to wound epithelialization, skin graft take rate, scar assessment, wound closure, and quality of skin structure.

#### Participant timeline {13}

**Fig. 4:**
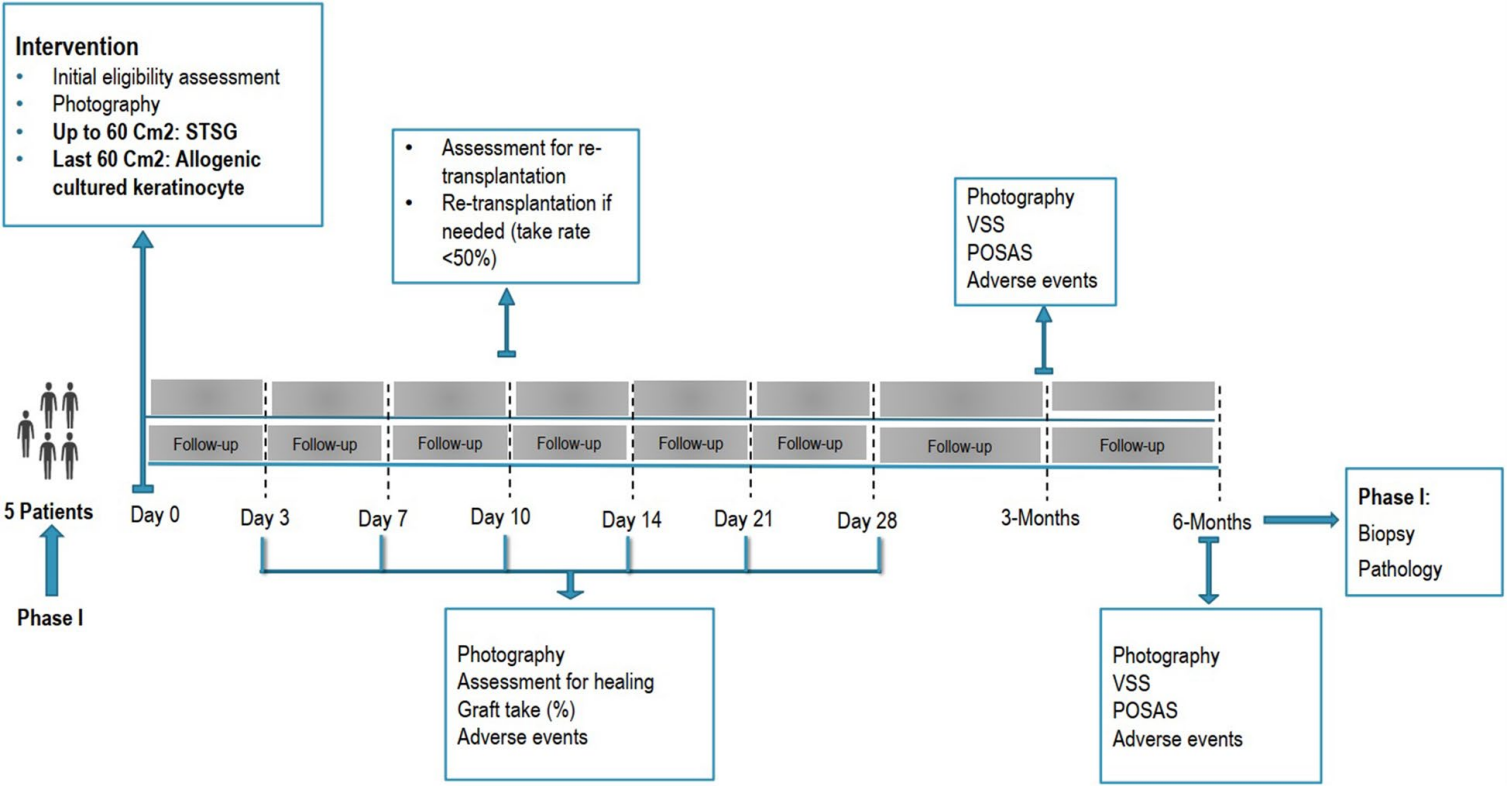
Follow-up timeline of the phase I study

**Fig. 5:**
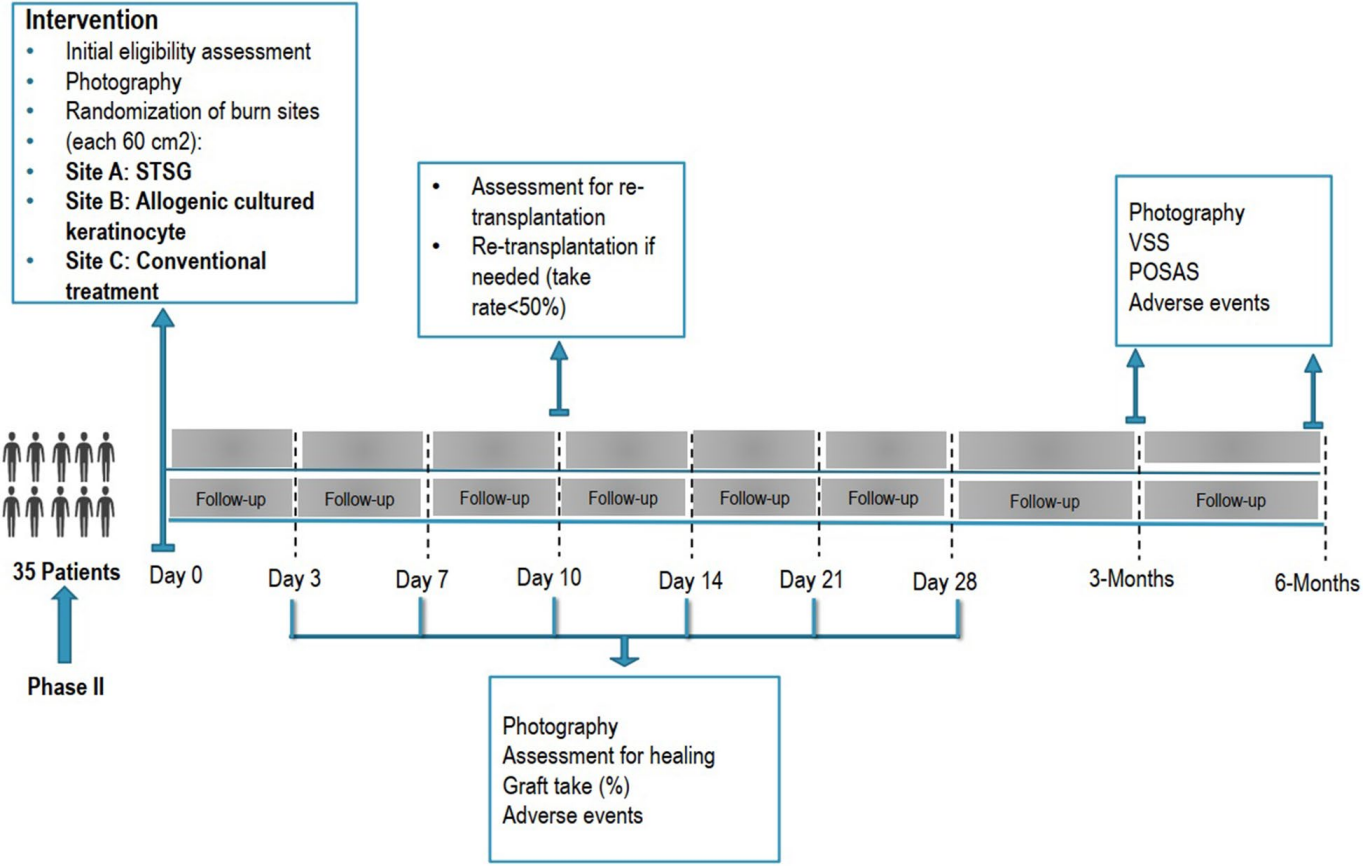
Follow-up timeline of the phase II study

The study timeline for phases I and II are presented in Figs. 4 and 5, respectively.

#### Sample size {14}

To determine the necessary sample size for the study comparing the response rate between the control group and the intervention group, the formula for duplicate sizes in the two groups was used. It was assumed that four rounds of transplantation would be performed, with a mean correlation of 0.5 between repetitive interpersonal measures, a 5% first type of error, a statistical power of 95%, and a minimum difference in response rate of 45% based on similar studies [26–29]. Using these parameters, a sample size of 40 patients was estimated. In the first phase of the study, 5 patients will be included and in the second phase, and 35 patients will be enrolled based on the approved inclusion and exclusion criteria.

$$n=\frac{{2\left( z_{\alpha/2}+ z_{\beta }\right)}^{2}{\sigma }^{2}\left\{1+\left(m-1\right)\rho \right\}}{{md}^{2}}$$

#### Recruitment {15}

Given that Shahid Motahari Hospital serves as a prominent referral center for burn cases in Iran, the considerable volume of patients meeting our specific inclusion criteria is noteworthy. As a result, the process of patient recruitment is anticipated to transpire within the projected timeframe.

### Assignment of interventions: allocation

#### Sequence generation {16a}

In phase I of the study, we will enroll eligible patients who meet the inclusion and exclusion criteria. This will be done through an open-label approach. In phase 2, random allocation software will be used as the method for randomization. By means of random allocation, each patient’s wounds will be assigned to one of the three groups: group A (autograft), group B (conventional treatment), or group C (keratinocyte sheet). This allocation will follow a 1:1:1 ratio and will adhere to a computer-generated randomization schedule that considers specific strata based on the wound site. Various wounds within a single patient will be subjected to this analysis. The investigation will involve a comparative analysis of the safety and efficacy of allogeneic skin grafts in contrast to autograft skin transplantation and conventional treatments, which encompass the use of Vaseline dressings and topical antibiotics.

#### Concealment mechanism {16b}

The mechanism of implementing the allocation sequence is central randomization. In this study, allocation of participants is performed centrally by a designated system (which generates a randomized list for allocation of patients in different groups) not directly involved in the recruitment or assessment of participants. Central randomization was implemented to ensure unbiased allocation of participants to treatment groups.

#### Implementation {16c}

The sequence of allocation will be conducted by an investigator (Dr. H.M.). Patients’ enrollment will be done by Dr. M.D. Assignment of patients to the groups will be done by Dr. S.F.

### Assignment of interventions: blinding

#### Who will be blinded {17a}

N/a: this study is open-label so there is no blinding.

#### Procedure for unblinding if needed {17b}

N/a: this study is open-label so there is no blinding.

### Data collection and management

#### Plans for assessment and collection of outcomes {18a}

Patients will be followed up on days 3, 7, 10, 14, 21, and 28, after the transplantation. In addition, in the 3rd and 6th months after the transplantation, we will also have additional follow-up sessions. In each follow-up session, a photo of the patient’s wound will be taken. The degree of epithelialization will be evaluated in each follow-up session through photography by the ImageJ software and at least three repetitive images for each wound will be taken. The skin graft take rate will be assessed on the 10th-day visit, and in the context of < 50% graft take, the allogenic transplantation will be repeated. In the 3rd and 6th month follow-ups, the scar and wound closure assessment will be conducted based on the Vancouver Scar Scale (VSS) and the Patient and Observer Scar Assessment Scale (POSAS).

#### Plans to promote participant retention and complete follow-up {18b}

##### Retention strategies

The study will implement a comprehensive retention strategy encompassing regular and frequent follow-up sessions. These follow-ups will involve diverse laboratory tests and complimentary visits. In addition, participants will receive written feedback regarding the outcomes of the conducted health screenings.

##### Withdrawal protocols

Participants hold the prerogative to withdraw from the study at their discretion, irrespective of the rationale, and at any juncture. Furthermore, the principal investigator retains the authority to withdraw participants from the study if it is deemed necessary for their safety or if they are unwilling or unable to adhere to the stipulated study protocols. The possibility of participant withdrawal also exists in the event that the study is prematurely terminated by the study sponsor, governmental bodies, or regulatory authorities prior to the planned culmination date. It should be noted that the discontinuation of the study product for any reason does not constitute grounds for withdrawal from the study.

#### Data management {19}

The data will be collected based on a research-made case report form (CRF). The CRF will include study timeline, history of present illness, basic laboratory data, CTCAE form, photographic and graft take rate information, standard questionnaires of VSS and POSAS, and also a form for pathological evaluation for phase one of the study.

#### Confidentiality {27}

All personal information of the patients will be collected and maintained by a dedicated investigator at Royan Institute in order to protect confidentiality.

#### Plans for collection, laboratory evaluation and storage of biological specimens for genetic or molecular analysis in this trial/future use {33}

In the phase 1 clinical study, 6 months after transplantation, a skin biopsy with a 2-mm^2^ diameter will be taken from three areas: areas treated with keratinocyte sheets, areas treated with autograft, and surrounding healthy skin. The samples will be dehydrated by formalin and ethanol, and after being placed in paraffin wax, they will be cut using a microtome with a thickness of 5 µm. Our samples will be stained with hematoxylin and eosin (H & E). The skin texture, skin ultrastructure, including hemidesmosomes, different tissue layers, and cellular infiltration will be assessed through microscopic evaluation. The collagen composition will be visualized by Masson’s Trichrome (MT) staining. In order to check the epithelialization, vascularization, fibrosis, and scarring, the immunohistochemical staining of tumor growth factor-beta (TGF-β) 1 and 3 will be performed. At least three images will be taken from each sample under the microscope. Due to the pathologic report and stored images, the samples will not be stored.

## Statistical methods

### Statistical methods for primary and secondary outcomes {20a}

After enrollment of all patients, the data will be entered into the SPSS software for statistical analysis. Based on the distribution of data, paired sample *t*-test or non-parametric equal (chi-square test) will be used for comparing the difference in before and after sample data. For comparing each group in phase II, a Student *t*-test or chi-square test will be administered. Moreover, to handle missing data, a multiple imputation test will be used.

### Interim analyses {21b}

In phase I, P2–P5 will receive a keratinocyte sheet with a 1-month interval after P1 for safety considerations. Moreover, all participants in phase I and II will be monitored at 3, 7, 10, 14, 21, and 28 days and 3 and 6 months after transplantation by consideration of any probable AEs and SAEs, and wound observation by the investigator. After the completion of the phase I clinical trial, all data includes AEs and SAEs, and wound healing process will be assessed and reviewed to confirm the safety of the process for starting the next phase. After the completion of the phase II clinical trial, all data from both phases will be analyzed.

### Methods for additional analyses (e.g., subgroup analyses) {20b}

N/a. In this study, we have not done any subgroup analysis due to the limited number of total patients.

### Methods in analysis to handle protocol non-adherence and any statistical methods to handle missing data {20c}

Multiple imputation includes the generation of multiple plausible values for compensation of missing data points, therewith preserving the uncertainty and variability associated with the missing data. Furthermore, sensitivity analyses will be conducted to evaluate the robustness of study outcomes to different assumptions about the missing data mechanism.

### Plans to give access to the full protocol, participant-level data and statistical code {31c}

All data generated during this study are included in this article. The results of this phase I and II trial will be published in scientific journals and will be available for researchers, clinicians, and patients.

### Oversight and monitoring

#### Composition of the coordinating center and trial steering committee {5d}

Principal investigator and research physician:

- Design of protocol and revisions
- Preparation of CRF [case report forms]
- Publication of study reports

Trial management committee (TMC):

- Practical advice for the investigators
- Randomization
- Data validation

Data manager:

- Data entry
- Data verification

Lead investigators:

- Patients’ recruitment, data collection, and completion of CRFs, doing the follow-up sessions

#### Composition of the data monitoring committee, its role and reporting structure {21a}

An independent Data and Safety Monitoring Board (DSMB) has been instituted, separate from the study coordinators. By the end of the phase 1 study, data analyses will be confidentially shared with the DSMB. Additionally, any supplementary analyses that the committee deems necessary will also be provided. These supplementary analyses might encompass data from similar trials for comparison. In this regard, the DSMB will advise the investigators.

#### Adverse event reporting and harms {22}

The primary endpoint for this study is safety, which will be evaluated in all follow-ups by Common Terminology Criteria for Adverse Event (CTCAE), version 5.0 [27]. All the data will be entered in CRF forms of the study.

#### Frequency and plans for auditing trial conduct {23}

The audit will assess the phase 1 clinical trial before the first visit of the first patient and after the final visit of the last patient. In phase 2, the audit will evaluate the study after enrollment of the 5th patients and 20th patients and at the end of the study.

#### Plans for communicating important protocol amendments to relevant parties (e.g., trial participants, ethical committees) {25}

Any alterations to the protocol that could influence the study’s implementation, potential patient advantage, or impact patient safety such as adjustments to study goals, design, participant demographics, sample sizes, study procedures, or substantial administrative elements will necessitate an official protocol amendment. Ethics Committee endorsement will be sought for such amendments.

#### Dissemination plans {31a}

All data generated during this study are included in this article. The results of this phase I and II trial will be published in scientific journals and will be available for researchers, clinicians, and patients.

## Discussion

In this study, we explained the design and rationale of a cellular-based skin substitute for the first time in Iran. It has been observed that almost £20,000 per quality-adjusted life year (QALY) is the annual cost of skin graft for 2nd-degree burn injuries [30]. Therefore, designing a substitute for skin grafting can help affected patients and reduces the invasive procedure of autologous skin graft. In addition, conducting this work proposes this product for being registered as an off-the-shelf product for burn wound management in the regulatory bodies of the country.

This project is novel to conduct in Iran; however, some limitations can exist in our work. First, many patients with 2nd-degree burn injuries may experience other trauma or co-existence morbidities, which can make the choice of eligible patients difficult for us. Second, due to its novelty, many patients may not collaborate to participate in this study. Third, many eligible patients may receive other treatment options, prior to transplantation, which can be a confounder for our intervention. We will address possible limitations in our first phase and will try to overcome our limitations in the second phase.

## Trial status

This is the protocol version 1 designed on 2022–04-25. Patients’ recruitment began on 7 November 2022. Patients’ recruitment will be completed on 30 November 2023, approximately.

## Data Availability

The results of both phase 1 and 2 studies will be published in scientific journals, and all necessary information will be available in the published manuscript and supplementary materials. Moreover, any extra information and datasheets will be available by sending a request to the principal investigator (Dr. M.V.).

## Abbreviations

IRCT: Iranian Registry of Clinical Trials
CRF: Case report form
QALY: Quality-adjusted life year
VSS: Vancouver Scar Scale
POSAS: Patient and Observer Scar Assessment Scale
DSMB: Data and Safety Monitoring Board
